# Theodor Bilharz’ 200th birthday

**DOI:** 10.1186/s40249-025-01359-9

**Published:** 2025-08-22

**Authors:** Joachim Richter

**Affiliations:** 1 Institute of International Health, Global Health Centre of Charité, Charité University Medicine, Corporate Member of Free University and Humboldt University, Berlin, 13353 Germany; 2 Swiss Tropical and Public Health Institute, Basel, Switzerland

## Abstract

**Supplementary Information:**

The online version contains supplementary material available at 10.1186/s40249-025-01359-9.

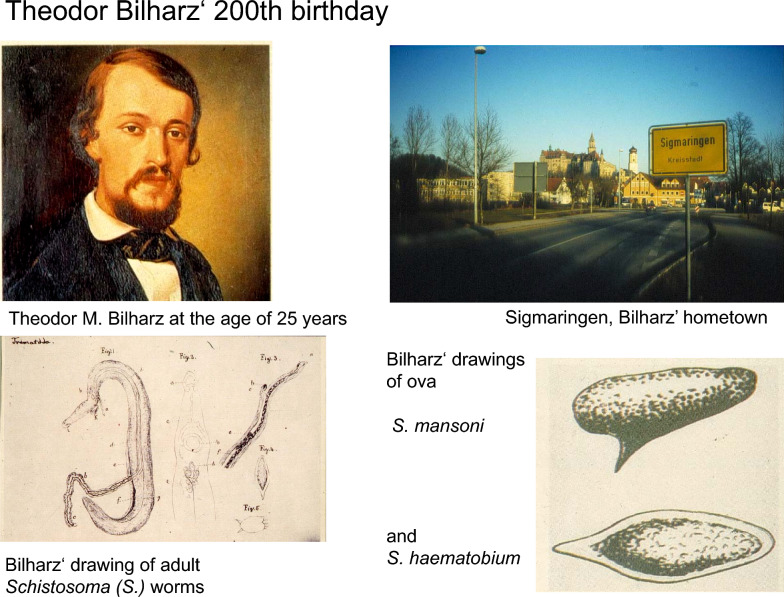

When you end up in a small town in the Danube valley in Southwest Germany you hardly imagine that an internationally well-known scientist grew up here: Theodor Maximilian Bilharz was born on March 23, 1825, in the town of Sigmaringen. By chance, 130 years later, I happened to be born in the same town. There, I went to school, which later took the name of “Bilharzschule”, there was a pharmacy called “Bilharzapotheke”, and I walked through “Bilharzstraße”. Like most citizens of Sigmaringen, I knew his name but had no idea what he stood for. How could I imagine that this man became famous for having discovered the cause of an important worldwide tropical disease known since antiquity, which is not even endemic in Germany? Why a son of a small town in the green Danube valley should have left his homeland for Egypt?

But he did so. He qualified in Medicine in 1849 at the nearby University of Tübingen. The former lecturer of Tübingen University, Wilhelm Griesinger, asked him to join him in Cairo. King Abbas Hilmi I had called European scientists to promote the modernization of his country. This is how the 25-year-old Theodor arrived in Egypt in 1850 [1]. After Griesinger left two years later, Theodor Bilharz was appointed head of the Medical Department of the Qasr el Einy hospital in Cairo and Director of the Cairo Medical School. He found himself confronted with a disease that had been known in Egypt since antiquity and was a particular scourge of the fellahin, the Egyptian peasants. Bilharz started with unencumbered enthusiasm to perform autopsies on corpses of persons who had died from “endemic hematuria”. Between 1851 and 1853, in a series of autopsies Bilharz found male and female adult trematode worms in the bladder wall and in the mesenteric veins (“Eingeweidewürmer”) as he reported in his letters to his former parasitology teacher in Freiburg University, Carl von Siebold, and named the new species: *“Distoma haematobium”* [1, 2]. Later, the genus *Bilharzia* was named after Theodor Bilharz, but it was ultimately renamed *Schistosoma haematobium*. Due to the name of these worms, the disease, which was previously known as bilharziosis, is now also commonly referred to as schistosomiasis in English and other languages.

Before the advent of microphotography, biologists had to be also graphic artists, as confirmed by the quality of Bilharz’s drawings of the adult worm pairs and the ova of the *“Schistosoma”* helminths (Fig. 1).

**Fig. 1:**
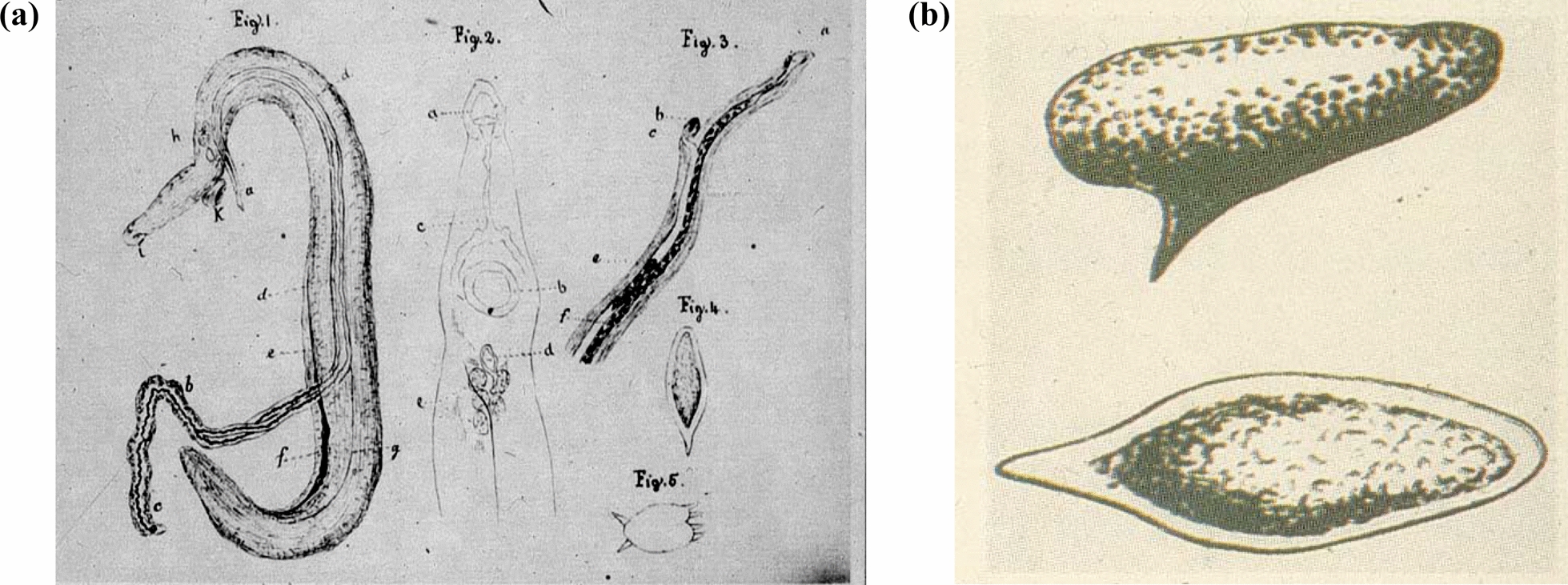
Original drawings by T. Bilharz: **a**. Adult worms and **b**. ova of schistosomes (*S. mansoni* and *S. haematobium*) [3]

During his studies Bilharz also discovered other worm species, now known as *Hymenolepis nana* and *Heterophyes heterophyes* and described the electric organ of Nile-electric catfish (*Malapterurus electricus*) as well as another Nile fish (*Brycinus macrolepidotus*) [1].

Theodor Bilharz died in 1862 at the age of 37 from an epidemic febrile infection, after serving as the personal doctor to Princess Alexandrine von Baden, who had contracted the illness during a journey with Duke Ernst II. von Sachsen-Coburg to the Valley of the Kings [1, 4].

The latter medical doctors were aware of the threat of epidemics of tropical diseases such as cholera, having been imported from the tropics to European cities such as London in 1854 and Hamburg in 1892, and schistosomiasis having infected troops returning from South Africa. Still not knowing the transmission modality of *Bilharzia*, they were afraid that schistosomiasis could be imported by the British colonial soldiers to the UK. Their fear was not unfounded, schistosomiasis having been endemic in Cyprus and later imported by Portuguese troops from today’s Moçambique and Angola to Southern Portugal. Interestingly, during Napoleon’s campaign in Egypt, French soldiers had been affected by “a most stubborn hematuria” as reported by the army surgeon AJ Renoult in 1798, but did not import the disease to France. In 2014, we discovered the first autochthonous cases of schistosomiasis transmitted in the French island of Corsica (where Napoleon had been born) to a German family [5].

Studies on the transmission cycle became more practicable after schistosomiasis had been discovered to be endemic also in Asia, and the species “*Bilharzia haematobium japonicum*” was described in Japan by Fujiro Katsurada (who had studied also at the University of Freiburg) and independently by Akira Fujinami. This discovery allowed for the performance of systematic animal studies in Asia. These studies confirmed the suspicion of percutaneous infection by *Bilharzia* worms. It took more than 60 years after Bilharz’s discovery of adult schistosome worms, their eggs, and miracidia before Robert Thomson Leiper succeeded to finally unravel the complete life cycle of schistosomes. He demonstrated that infection did not occur via the oral route, but rather through skin penetration by fork-tailed larvae (furcocercaria) that developed in freshwater snails – an obligatory intermediate host. Leiper also confirmed that the terminal- and lateral-spined eggs first illustrated by Theodor Bilharz belonged to two distinct species: *S. haematobium* and *S. mansoni* (Fig. 1.) [1]. In 1918, while treating patients with leishmaniasis, Christopherson discovered that tartar-emetic was also effective against concomitant schistosomiasis. Finally, it took more than another 50 years for the development of well-tolerated oral drugs such as metrifonate, oxamniquine and, later, praziquantel, enabling the envisage a successful control in regions with limited resources [1, 3]. Today, more than 170 years after Theodor Bilharz’s discovery, schistosomiasis still affects over 5% of the population in some Egyptian governorates, underscoring the ongoing need for sustained control efforts [6]. The WHO reports that, although the global schistosomiasis burden has decreased significantly over the past 30 years, transmission still occurs in 78 countries, with at least 251.4 million people requiring preventive treatment in 2021.

## Supplementary Information

Supplementary Material 1.

## Data Availability

Supportive figures are provided.
