# Supplementary material for: Theodor Bilharz’ 200th birthday
Source: Infect Dis Poverty. 2025 Aug 22;14:89. doi: 10.1186/s40249-025-01359-9 (PMC12372399; doi:10.1186/s40249-025-01359-9)
Supplement: Supplementary file 1 — Supplementary Material 1. [file 40249_2025_1359_MOESM1_ESM.docx]

**Annex**

**200 anniversary of Theodor Bilharz‘ birthday**


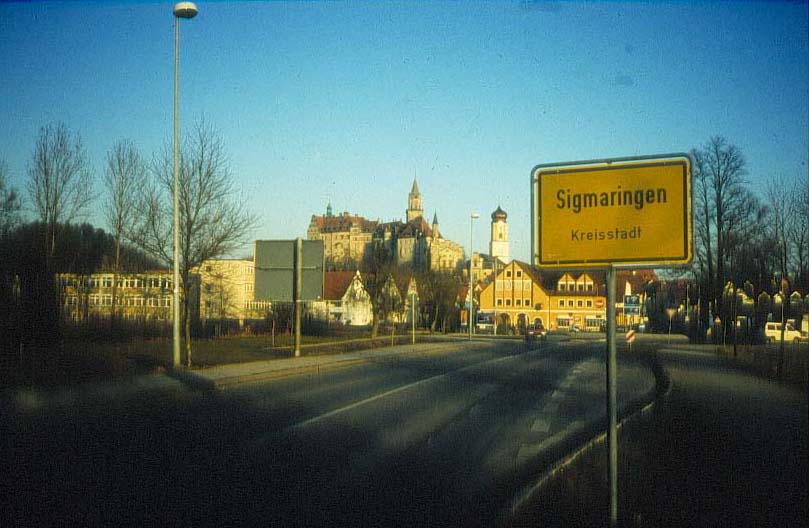


Entry to town of Sigmaringen with castle and church tower.


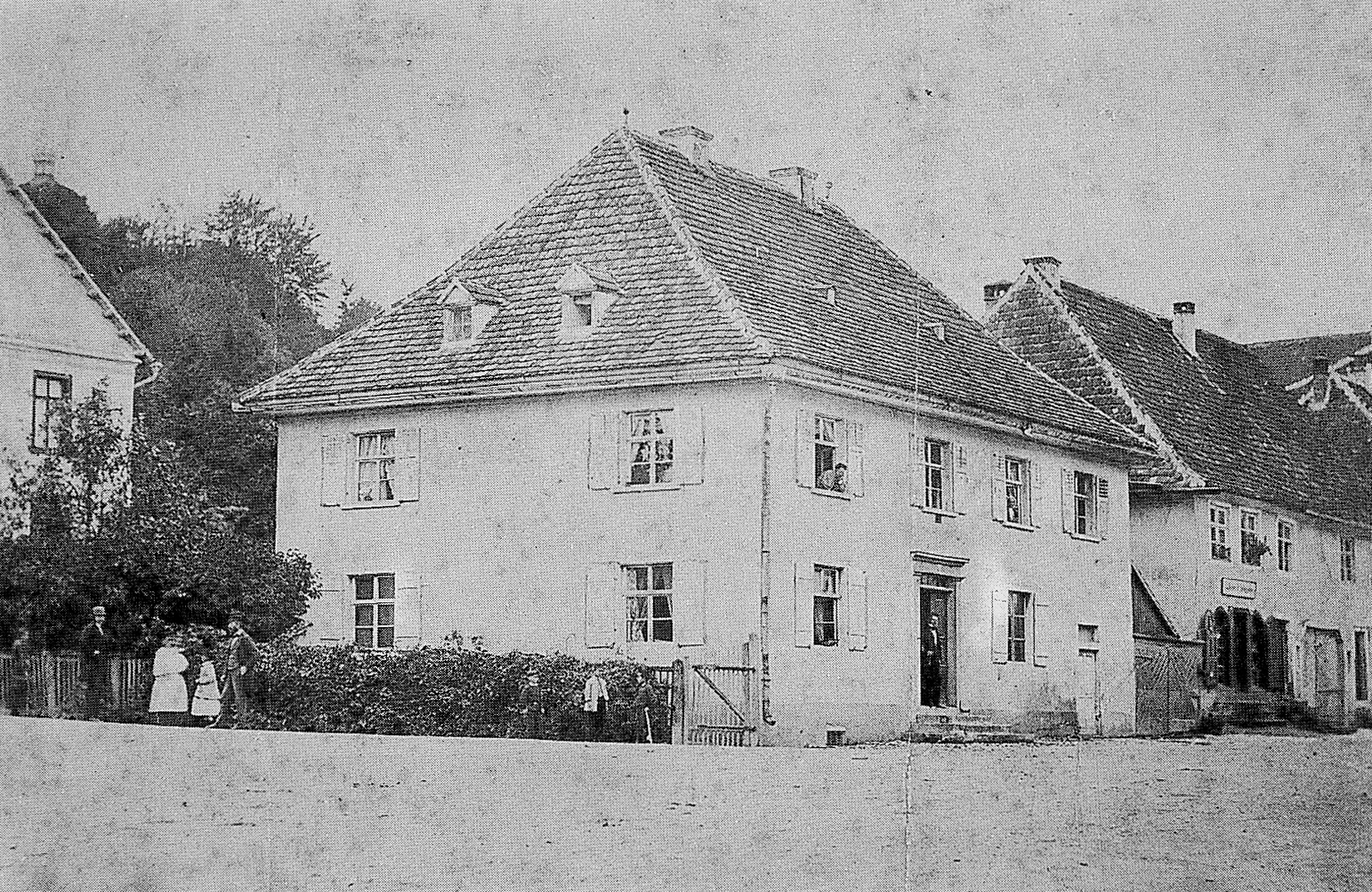

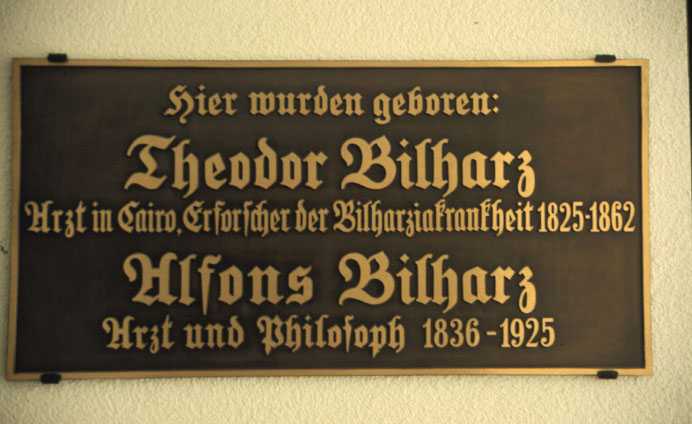


House where Theodor and his siblings were born in Sigmaringen. Today a table inside the house remembers him and his younger brother Alfons, who founded the Sigmaringen hospital


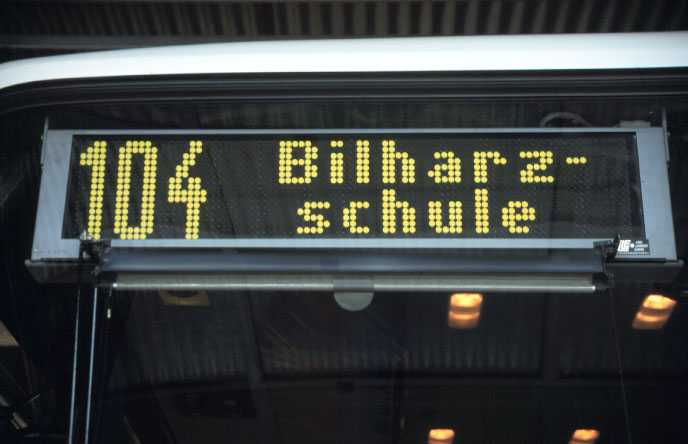


Bus to school “Bilharzschule” in Sigmaringen


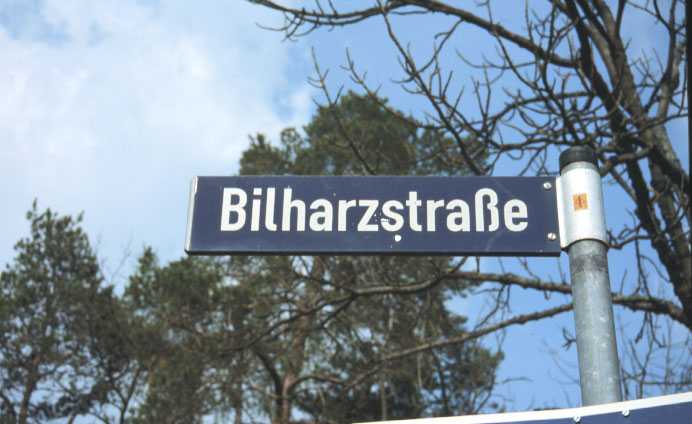


“Bilharzstraße” in Sigmaringen


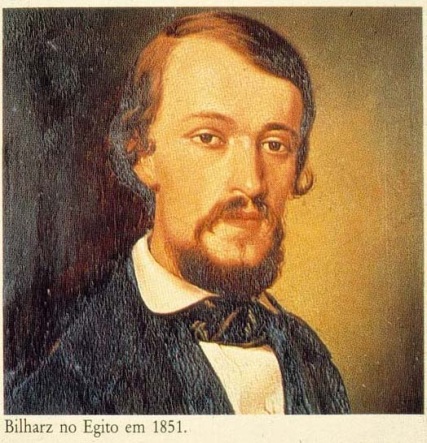


Portrait of Theodor Bilharz at the age of 25 depicted by an anymous artist in Cairo


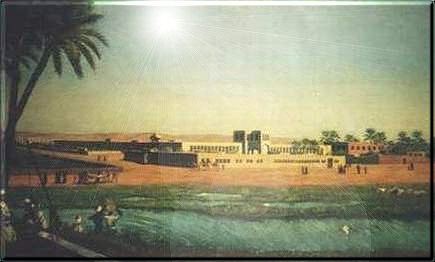


Qasr el Eyni hospital in Cairo


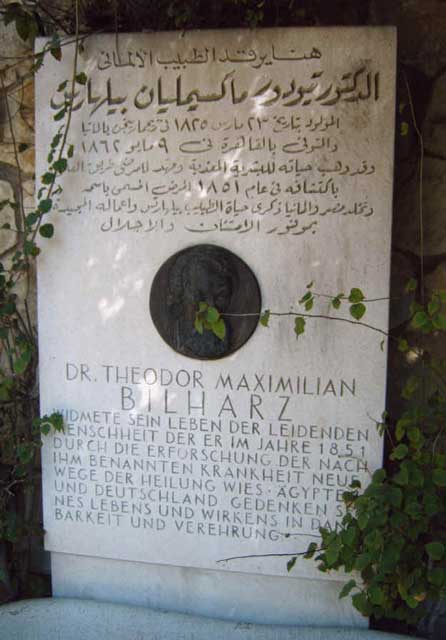


Bilharz’ grave in Cairo.


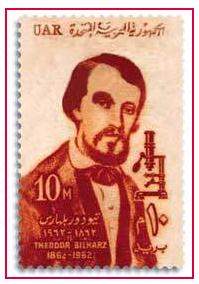


Eygptian stamp. Theodor Bilharz


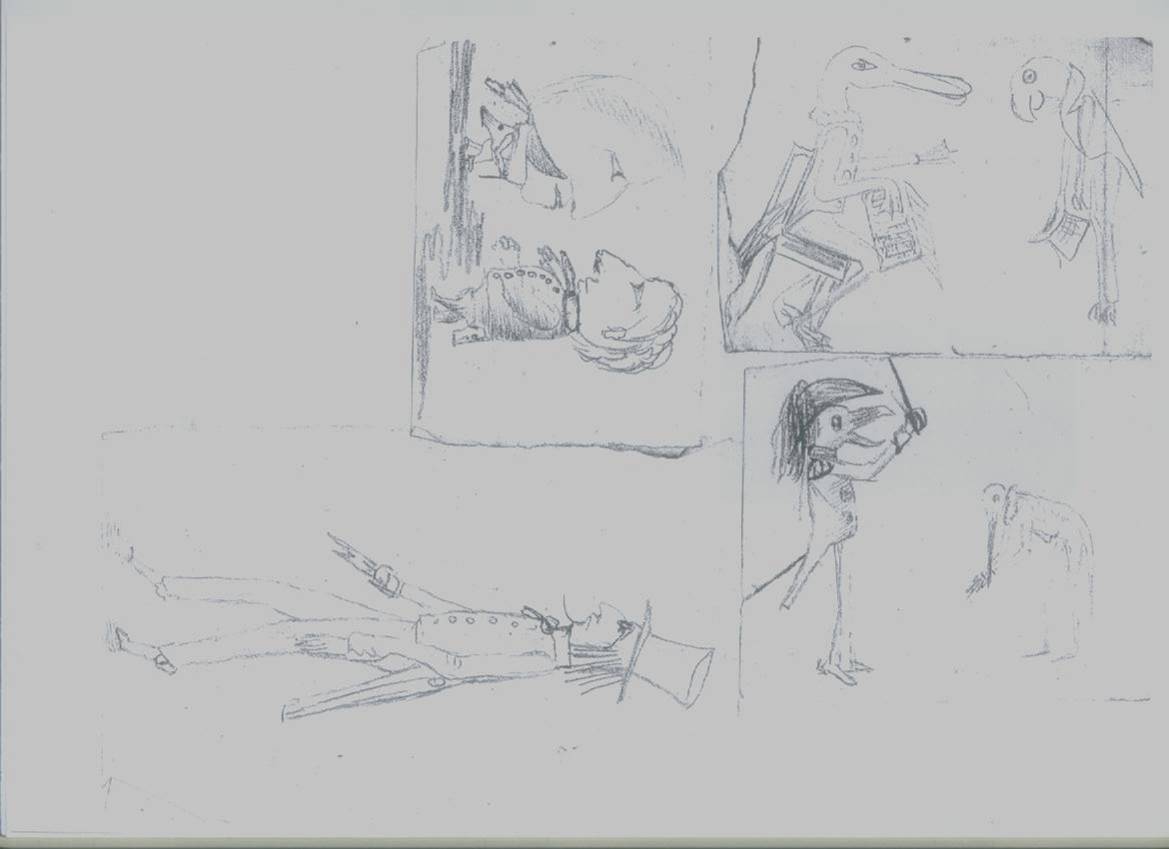

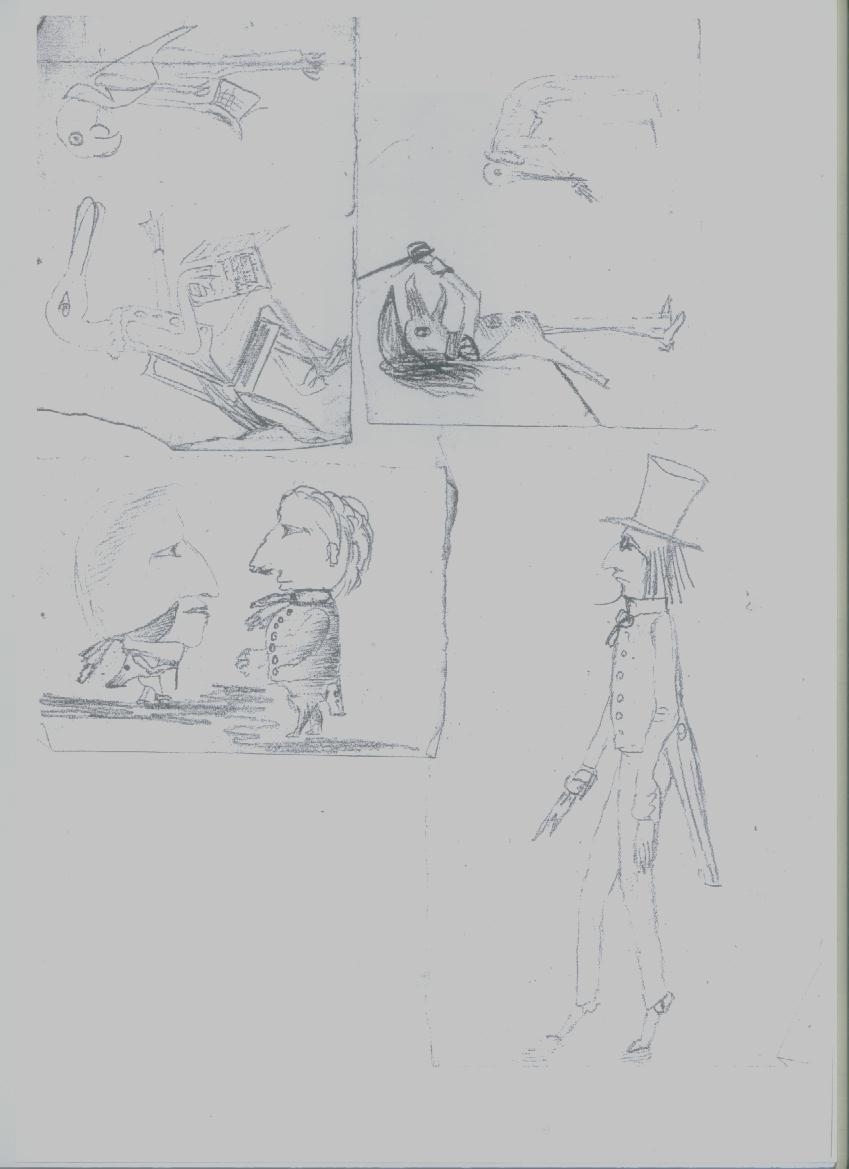


Ironic drawings depicted in school by the young Theodor Bilharz, Institute of History of Medicine, Heinrich-Heine-University, Düsseldorf, Germany

1. **Further references**
2. Bilharz, Theodor. Das electrische Organ des Zitterwelses. Anatomisch beschrieben. Mit 4 kolor. lithogr. Tafeln. Leipzig, W. Engelmann 1857.
3. Farley, John. Bilharzia: a history of imperial tropical medicine. Cambridge History of Medicine, UK. 1993; first paperback edition 2003.
4. Nevill, Anneli, Bilharziose oder Schistosomiasis. Geschichte, Epidemiologie und Pathologie einer Tropenkrankheit. Basilisken Presse Rangsdorf, Germany, 2010.
5. Albrecht, Helmuth. Schwäbische Forscher und Gelehrte. DRW Verlag Stuttgart, Germany 1992. Pp. 98-102.
6. Sturrock RF. Discovery. In Jordan P, Surrock RF & Webbe G. Human Schistosomiasis. CAB International, Wallingford UK, 1993; chapter 1. Pp. 1-4.
